# Temporal Evolution of the Gravitaxis of *Euglena gracilis* from a Single Cell

**DOI:** 10.3390/plants10071411

**Published:** 2021-07-09

**Authors:** Kazunari Ozasa, Hyunwoong Kang, Simon Song, Shota Kato, Tomoko Shinomura, Mizuo Maeda

**Affiliations:** 1 Bioengineering Laboratory, Cluster for Pioneering Research, RIKEN, 2-1 Hirosawa, Wako, Saitama 351-0198, Japan; mizuo@riken.jp; 2 Advanced Laser Processing Research Team, RIKEN Center for Advanced Photonics, RIKEN, 2-1 Hirosawa, Wako, Saitama 351-0198, Japan; 3 Department of Mechanical Engineering, Hanyang University, 222 Wangsimni-ro, Seongdong-gu, Seoul 04763, Korea; kang1026@hanyang.ac.kr (H.K.); simonsong@hanyang.ac.kr (S.S.); 4 Institute of Nano Science and Technology, Hanyang University, 222 Wangsimni-ro, Seongdong-gu, Seoul 04763, Korea; 5 Center for Bioscience Research and Education, Utsunomiya University, Mine 350, Utsunomiya, Tochigi 321-8505, Japan; shota.kato.680@gmail.com; 6 Plant Molecular and Cellular Biology Laboratory, Department of Biosciences, School of Science and Engineering, Teikyo University, 1-1 Toyosatodai, Utsunomiya, Tochigi 320-8551, Japan; shinomura@nasu.bio.teikyo-u.ac.jp; 7 Liver Cancer Prevention Research Unit, Cluster for Pioneering Research, RIKEN, 2-1 Hirosawa, Wako, Saitama 351-01, Japan

**Keywords:** positive gravitaxis, microfluidic devices, cell metabolism, swimming traces, bioconvection

## Abstract

Gravitaxis is one of the most important issues in the growth of microalgae in the water column; it determines how easily cells receive sunlight with a comfortable intensity that is below the damaging threshold. We quantitatively investigated and analyzed the gravitaxis and cell multiplication of *Euglena gracilis* using vertically placed microchambers containing a single cell. A temporal change in gravitaxis and cell multiplication was observed after transferring the cells to fresh culture medium for 9 days. We performed 29 individual experiments with 2.5 mm × 2.5 mm × 0.1 mm square microchambers and found that the cells showed positive, negative, and moderate gravitaxis in 8, 7, and 14 cases, respectively, after transferring to fresh culture medium. A common trend was observed for the temporal change in gravitaxis for the eight initially positive gravitaxis cases. The cells with initially positive gravitaxis showed a higher rate of cell multiplication than those with initially negative gravitaxis. We also discussed the gravitaxis mechanism of *E. gracilis* from the observed trend of gravitaxis change and swimming traces. In addition, bioconvection in a larger and thicker chamber was investigated at a millimeter scale and visualized.

## 1. Introduction

Gravitaxis is a fundamental characteristic of both land and sea plants. Even in the absence of light, gravity indicates the direction in which plants should grow to obtain sunlight. A plant seed lying in soil can respond to gravity to determine the direction to extend its roots and shoots. Several motile microalgae in water also exhibit negative gravitaxis and gather near the surface area of the water column to catch sunlight [1,2,3]. Microalgae swimming in water may find it difficult to detect gravity, because buoyancy and water flow disturb gravity sensing [3]. For microalgae in water, one of the simplest ways to swim toward or against gravity is to use buoyancy, i.e., rising to the surface of the water column and sinking to the bottom by buoyancy-affected heading mechanisms [4,5]. In such cases of passive gravitaxis, a microalgal cell must change its center of mass from the anterior region to the stern region, or vice versa, to produce positive or negative gravitaxis [6,7]. The other approach to exhibit gravitaxis is for algae to detect the direction of gravity and swim upward/downward using its own propelling force. In this case, the microalgae can determine the sign of gravitaxis as per its age or growth stage, as well as from the environmental conditions, including nutrients and light. Hence, the gravitaxis of microalgae is a key factor in the industrial application of microalgae for green chemistry [8,9]. In addition, the phenomenon is important for the fishery and water leisure industries because natural microalgae blooms have a significant impact on sea and lake ecosystems [10,11].

Gravitaxis of unicellular microalgae has been extensively investigated, especially for *Chlamydomonas reinhardtii* and *Euglena gracilis*, both of which are eukaryotes with photosynthesizing chloroplasts and propelling devices. *C. reinhardtii* is a model organism for unicellular microalgal studies, possessing two anterior flagella and multiplying with both asexual and sexual reproduction. *E. gracilis* is also a model organism, possessing an emergent flagellum and multiplying only with asexual reproduction, i.e., cell division. *E. gracilis* is more preferable than *C. reinhardtii* for the investigation of the succession of characteristics such as gravitaxis and phototaxis, since no crossover and mixing of genotypes is involved in asexual reproduction of *E. gracilis*. Cellular structures of *E. gracilis* can be found in previous literatures with detailed microphotographs and electron micrographs [12,13].

We aimed to understand the way in which *E. gracilis* gravitaxis changes according to cell growth, multiplication, and chemical changes in a culture medium, as well as environmental changes in a water column. Previous studies [14,15] indicated that *E. gracilis* gravitaxis changes with cell culture conditions. Cells in a young culture exhibited positive gravitaxis, whereas those in an aged culture exhibited negative gravitaxis. We were also interested in the behavior and mechanism of gravitaxis, including the ways in which a cell changes its swimming direction with respect to gravity [16].

In this paper, we report the gravitactic behavior of a single *E. gracilis* cell and its temporal changes with cell multiplication. We examined three types of a micrometer scale water column chamber: a square microchamber to observe the entire area in situ, a rectangular microchamber to observe the top and bottom areas separately, and a mini test tube chamber to observe convection at the surface and bottom areas separately. Temporal changes in gravitaxis along with cell multiplication were measured from a single cell up to several hundreds of cells within 9 days. Results revealed that the gravitaxis of *E. gracilis* after being transferred to fresh culture medium was positive in ca. 28% of cases, negative in ca. 24%, and moderate in ca. 48%; however, all cases showed negative gravitaxis after 4 days of cell culture. In addition, we tracked the gravitactic motion of *E. gracilis* and discuss the mechanism of the gravitaxis of *E. gracilis* compared with its phototactic motion. Finally, the occurrence of bioconvection was investigated and visualized in a larger chamber at a millimeter scale.

## 2. Materials and Methods

*E. gracilis* Z strain cells were used in this study. The cells were cultured in CM medium [17] with the addition of 0.1% ethanol to assist cell growth via heterotrophical nutrition supply. The cells were maintained at the growth saturation level under moderate room light and room temperature (23 °C–26 °C) without air bubbling. The cell suspension was refreshed by successive cultures every two weeks. Approximately 2 h prior to starting the experiments, the cell suspension was diluted with fresh CM medium with 0.1% ethanol to a concentration of ca. 1000–5000 cells/mL. The pH value of the 2-week-old cell suspension was 4.62, whereas that of the fresh CM medium was 5.95. Therefore, the transfer to the fresh medium represented chemical environmental changes for the cells.

**Figure 1:**
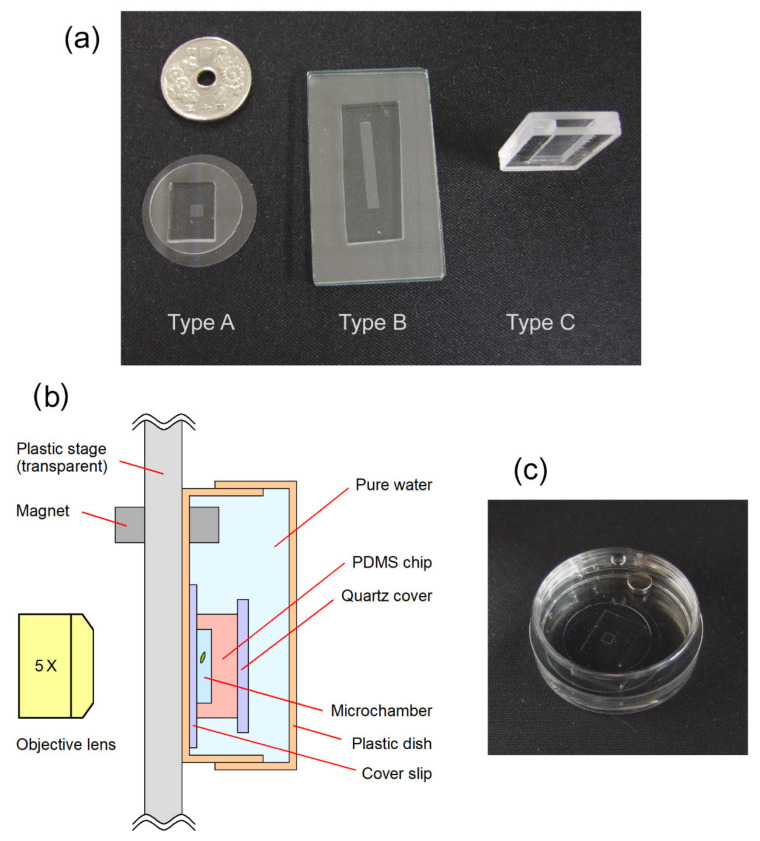
(**a**) Three types of microchamber used in this study. (**b**) Configuration of gravitaxis observation with the type A microchamber. Type A and B microchambers were contained in a plastic dish filled with pure water to prevent the microchamber from drying. (**c**) Photograph of the type A microchamber contained in a plastic dish filled with pure water, corresponding to the illustration (**b**).

We used three types of water column microchambers named A, B, and C. Type A was a square microchamber that was 2.5 mm in height (gravity direction) × 2.5 mm in width × 100 μm in thickness. Type B was a rectangular microchamber that was 20 mm in height × 2.5 mm in width × 100 μm in thickness. Type C was a mini test tube chamber that was 20 mm in height × 8 mm in width × 1.6 mm in thickness. The liquid holding capacity of type A, B, and C microchambers was 0.625, 5.0, and 256 μL, respectively. All areas of the type A microchamber were observed in situ in a single frame of our optical microscope, whereas only the top and bottom areas were separately observed for types B and C microchambers. Types A and B microchambers were made up of polydimethylsiloxane (PDMS) and covered with a glass coverslip after placing a droplet of *E. gracilis* cell suspension on the microchamber. The cell concentration of the suspension was low enough that only a single cell could be confined in the type A and B microchambers after a couple of trials. The type C chamber was an acrylic cell with an open top. For experiments using the type C chamber, the chamber was first filled with fresh CM medium with 0.1% ethanol and, subsequently, 10 μL cell suspension was added to the medium; this resulted in ca. 100–500 cells in the chamber. Figure 1 shows the outlook of each microchamber.

We conducted 29, 5, and 6 experiments with type A, B, and C microchambers, respectively. Three to seven experiments with individual type A microchambers were undertaken simultaneously, six times in total. The experiments with type B and C microchambers were performed one by one. The cells for the experiments were taken from the two-week-old cell suspension and transferred to a fresh CM medium with 0.1% ethanol to maintain the initial cell condition of each experiment as closely as possible. A vertical microscope with a 5X objective lens (MPlanFLN 5X/0.15, Olympus, Tokyo, Japan) was used to observe the vertically placed microchambers [18]. The experiments were performed under room illumination at room temperature, and infrared illumination was used for microscopic observation. As we previously reported [19,20], cell movement was tracked using video image processing technology developed in-house. The images were 800 × 600 pixels, with a resolution of 2500 μm/512 pixels, and refreshed at a frame rate of ca. 12.7 s/frame. The swimming traces in each image were counted using pixels and named “trace momentum (TM)” to represent the total swimming activity of the cells within the observation area. For type A experiments, the center of gravity of swimming traces (Xc, Yc) was calculated to estimate the gravitaxis of the cells. For the larger microchambers of types B and C, only the top and bottom areas were separately observed.

## 3. Results

### 3.1. Temporal Evolution of Cell Multiplication and Gravitaxis

**Figure 2:**
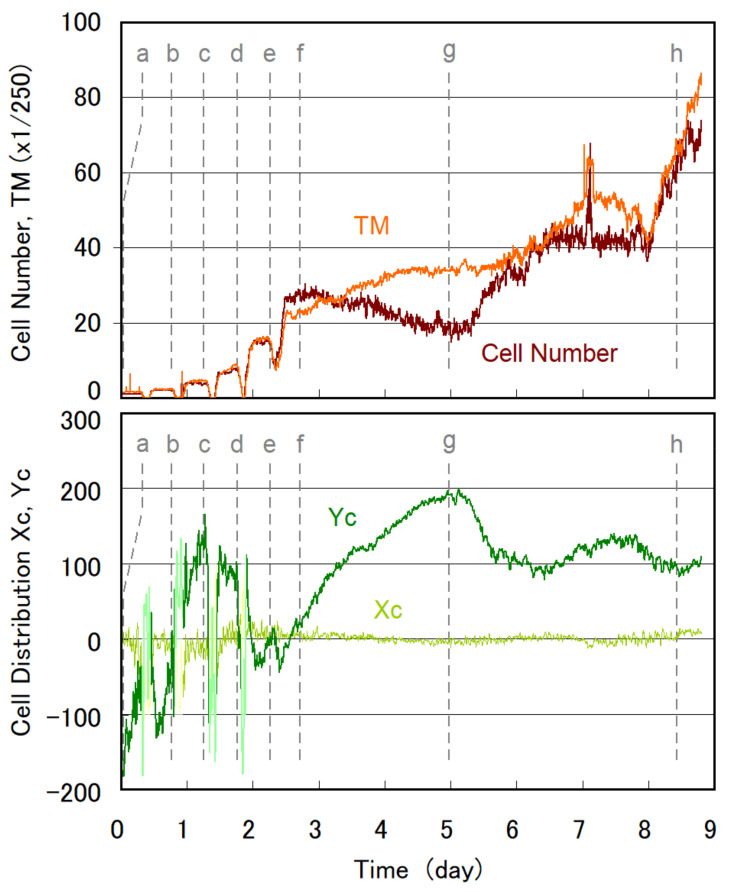
Temporal change in the center of cell distribution (Xc, Yc), swimming activity evaluated as a TM value, and cell numbers estimated by our analyzing algorithm. Cell distribution (Xc, Yc) was not valid for the periods of no TM value and is therefore faded in the figure. Irregular notches were due to some artifacts, such as noise and unintentional microchamber movements. The early stage of day 0.0–2.2 is presented in Figure S1.

**Figure 3:**
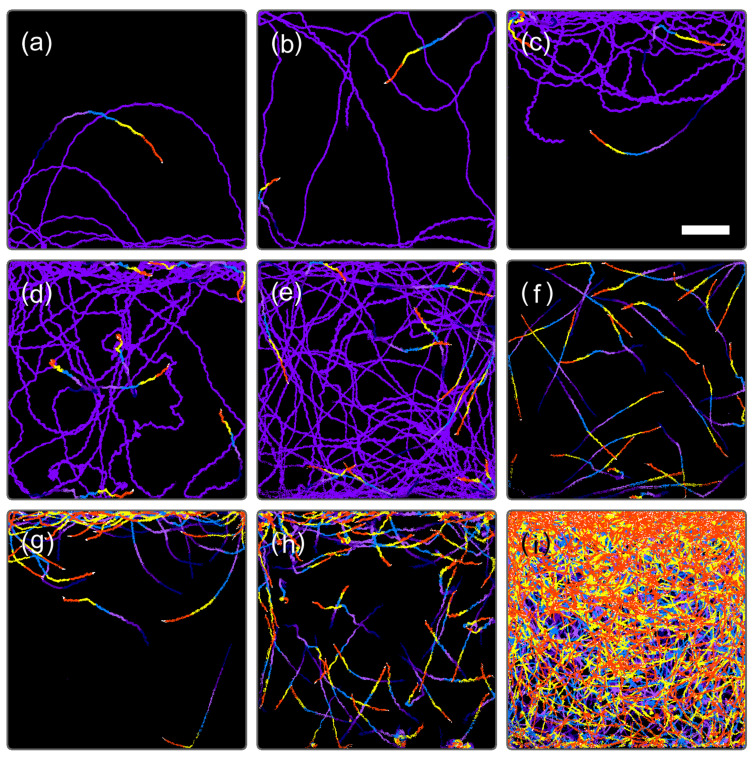
Trace image observed at day (**a**) 0.01, (**b**) 0.77, (**c**) 1.26, (**d**) 1.76, (**e**) 2.26, (**f**) 2.72, (**g**) 4.97, (**h**) 8.44, and (**i**) 13.97, corresponding to the timings indicated in Figure 2. Traces for 149.2 and 12.4 s were superimposed for (**a**–**e**) and (**f**–**i**), respectively. In the images of (**a**–**i**), traces obtained for the last 12.4 s (6 frames × 2.07 s/frame) were superimposed with different colors for visualization purposes. Scale bar in (**c**) indicates 0.5 mm.

Figure 2 shows an example of the temporal evolution of the center of cell distribution (Xc, Yc), swimming activity evaluated as a TM value, and cell number estimated by our algorithm previously reported [21]. The detail of the initial stage of Figure 2 is presented as an expanded plot in Supplementary Figure S1. Trace images are shown in Figure 3 for the representative timings indicated in Figure 2. The center of the microchamber corresponds to (Xc, Yc) = (0.0, 0.0), whereas the top and bottom edges correspond to Yc = 256 and −256, respectively.

At the early stage (day 0.0–0.06, 0.0–1.4 h) of cell culture in the type A microchamber, the Yc value was negative and ranged from −180 to −150. This indicates that the cell stayed within 520 μm of the bottom edge of the microchamber, an area in the lowest quarter of the water column. The swimming traces presented in Figure 3a showed that the cell exhibited positive gravitaxis, with a circular swimming trace toward the bottom of the microchamber. The positive gravitaxis of the cell was gradually weakened up to Yc = −35 for 1.0–7.4 h (day 0.04–0.31). The TM value remained nearly constant in this period, indicating that the swimming speed of the cell did not significantly change. The swimming trace of the cell gradually changed from circular to straight swimming.

The first cell division occurred at 7.7 h (day 0.32) (Figure 2 and Figure S1), where the decrease in the TM value showed that the cell ceased swimming. At cell division, the flagellum of *E. gracilis* is degraded to zero length and the cell usually sinks to the bottom of the water column [12,22,23,24]. The cell in Figure 3a also sank down and divided into two daughter cells. Because the cell did not move during cell division, the TM value dropped to zero, and the Yc and Xc values became unreliable because of the lack of trace images of the cell. Two new daughter cells started swimming at 10.6 h (day 0.44) and initially showed almost no gravitaxis (Yc was close to zero). After 30 min, a shift to positive gravitaxis occurred, and Yc became nearly −120 at 14 h (day 0.58). After 14 h (day 0.6), gravitaxis gradually shifted to negative from Yc = −120 to zero at 18.5 h (day 0.77) and the cells swam without directional preferences (Figure 3b). After the second cell division at 20.0–22.6 h (day 0.83–0.94), the gravitaxis of four cells became strongly negative (Yc was close to 130) and the cells stayed in the uppermost third area (Figure 3c). After the third cell division at day 1.35–1.43, seven or eight cells swam in a larger area (Figure 3d) than observed previously (Figure 3c), indicating that a diversity of cell characteristics emerged. However, Yc remained close to 130, indicating strongly negative gravitaxis.

As the cell number increased to 16–30 for day 2.0–3.0, the Yc value returned to almost zero and swimming traces were distributed almost uniformly in the microchamber (Figure 3e,f). Subsequently, Yc gradually increased to ca. 200, indicating a highly negative gravitaxis with swimming traces remaining at the upper part of the microchamber (Figure 3g). Eventually, Yc settled at 70–130 after day 6.0, where some cells stayed in the upper part and some cells swam down to the bottom; however, they still showed circular swimming towards the top (Figure 3h).

The cell number estimated by our algorithm corresponded well with the TM value, indicating that the value represents the progress of cell multiplication. The deviations observed at day 4.0–5.6 were due to the underestimation of cell numbers owing to the overlap of swimming traces in trace images. Neither the cell number nor the TM value showed an ideal exponential growth, stagnating at day 3.0–8.0. The reason for the stagnant period is unclear.

### 3.2. Trends of Gravitaxis and Cell Multiplication

**Figure 4:**
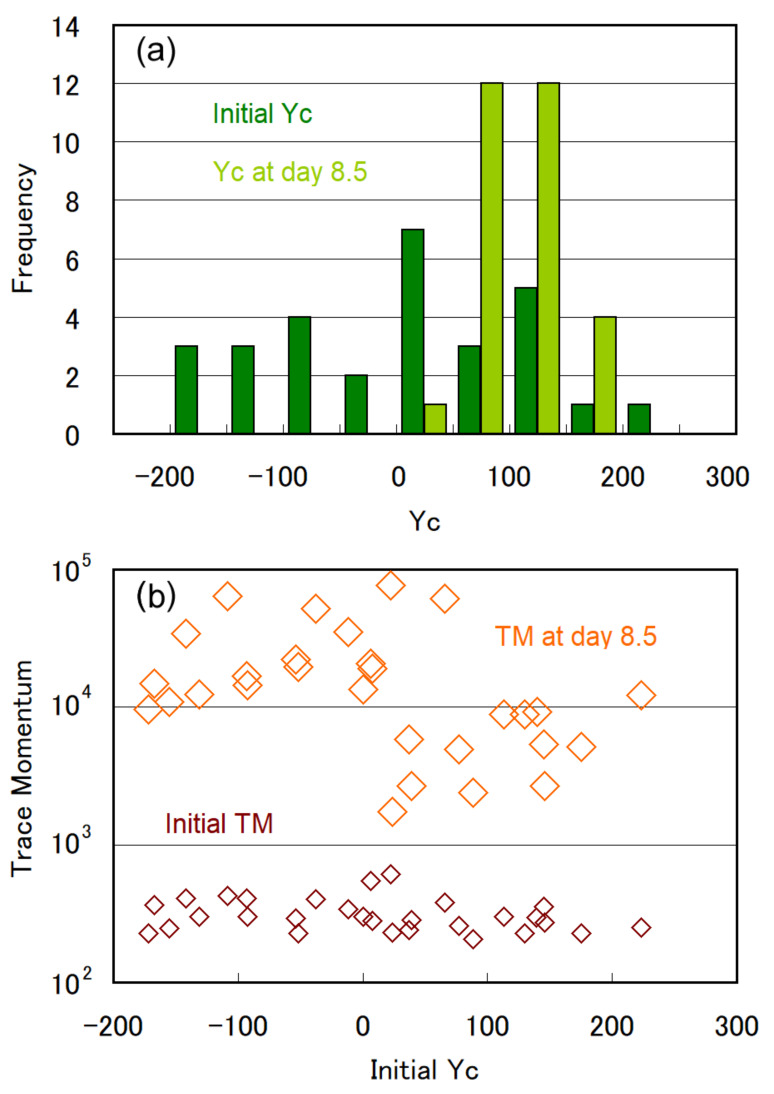
(**a**) Histogram of initial Yc and Yc at day 8.5, binned in 50 pixels. (**b**) Relation between initial Yc and trace momentum (initial TM and TM at day 8.5).

We performed 29 experiments with type A microchambers and found diversity in gravitaxis while focusing on the initial stage of cell multiplication. The histogram of “initial Yc” and “Yc at day 8.5 ” is shown in Figure 4a. We categorized 29 experiments according to initial Yc as positive (Yc < −90), negative (Yc > 90), or moderate (−90 < Yc < 90) for initial gravitaxis. The categorization resulted in 8 positive, 7 negative, and 14 moderate initial gravitaxis. Approximately 30% of the *E. gracilis* cells that were transferred to fresh CM medium exhibited positive gravitaxis.

**Figure 5:**
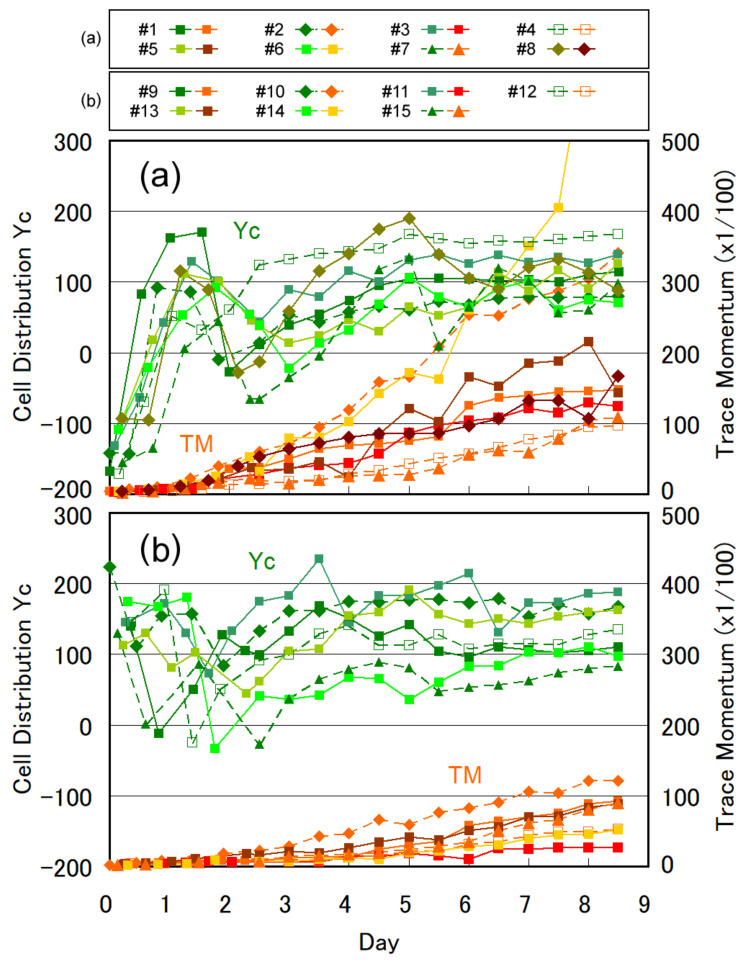
Temporal change in the center of cell distribution Yc and swimming activity evaluated as a TM value for (**a**) 8 cases of initially positive gravitaxis and (**b**) 7 cases of initially negative gravitaxis. See Supplementary Figure S2 for 14 cases of initially moderate gravitaxis. Case 8 in Figure 4a corresponds to Figure 2.

The temporal change in the Yc and TM values are plotted in Figure 5a,b for 8 positive and 7 negative cases. The same plot for 14 moderate cases is presented in Supplementary Figure S2. Although the plots show a natural deviation and scattering owing to the diversity in cell characteristics, interesting trends can be deduced from the data shown in Figure 5a. For the initially positive cases in Figure 5a, the gravitaxis became negative when the cell number increased from four to eight and subsequently became moderate at day 2–3. The cells showed negative gravitaxis again after day 4, and Yc eventually converged to a range of 100 ± 50. Of note, this trend was observed for all eight initially positive cases (Figure 5a). This observation showed that the changes in gravitaxis were mostly synchronized after the transfer of the cells to fresh CM medium. The temporal change in Yc did not show specific trends for the initially negative cases shown in Figure 5b nor the initially moderate cases shown in Figure S2, except that Yc converged in the range of 100 ± 80. The convergence of Yc with cell multiplication can be clearly observed in Figure 4a in the histogram of Yc at day 8.5.

The rate of cell multiplication observed in the experiments was also diverse, as per the TM growth (Figure 5a,b and Figure S2). Some cases showed an ideal exponential growth, including cases 6, 18, and 29, whereas some were nearly saturated, such as 11 and 16. However, we found a significant relationship between the cell multiplication rate and initial gravitaxis in the plot of TM value versus initial Yc (Figure 4b). Although the initial TM values at the early stage of cell culture were almost independent of the initial Yc, the TM values at day 8.5were higher for the cases with Yc < 0 (positive gravitaxis) than for those with Yc > 0 (negative gravitaxis).

### 3.3. Specific Observation for Top and Bottom Area

**Figure 6:**
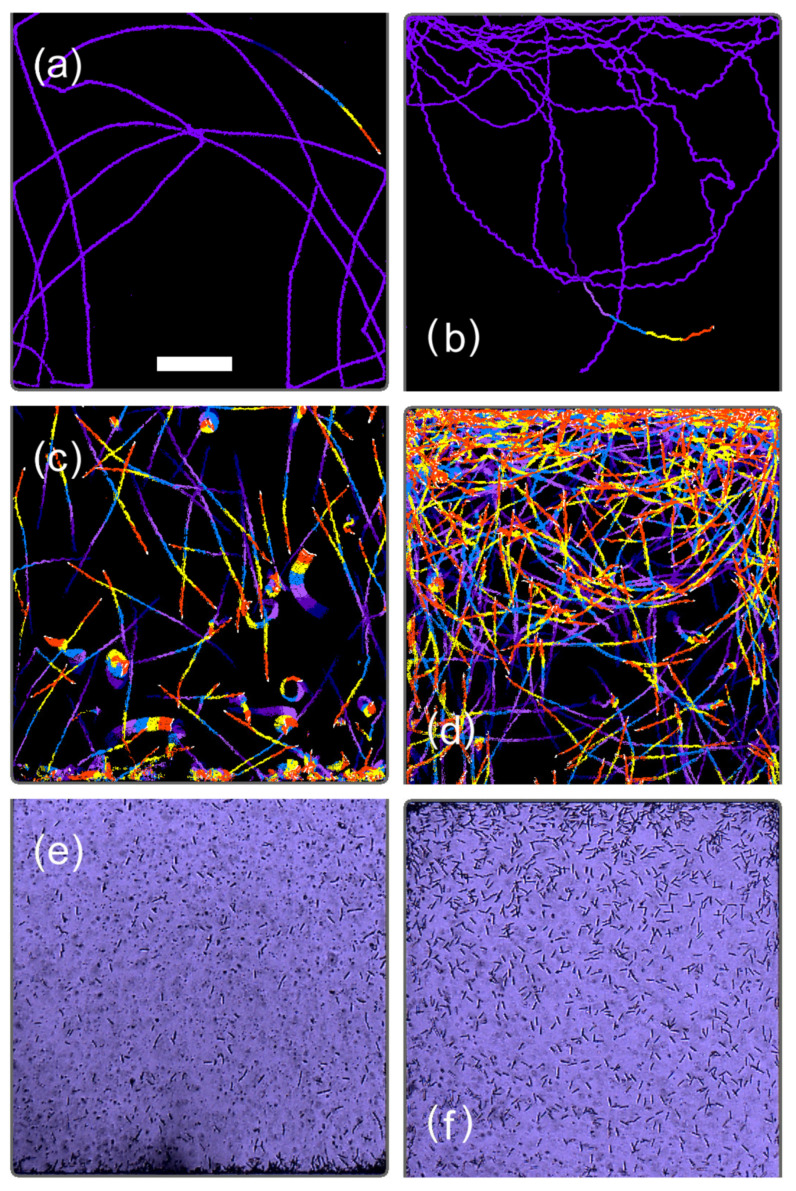
Trace images (**a**–**d**) showing cellular movements and a real image (**e**,**f**) of *E. gracilis* observed for type B experiment. (**a**,**c**,**e**) Bottom area, (**b**,**d**,**f**) top area at (**a**) 0.96 h (day 0.04), (**b**) 18.7 h (day 0.78), (**c**,**d**) day 8.32, and (**e**,**f**) day 11.32. Traces for 402.0 and 12.6 s were superimposed for (**a**,**b**) and (**c**,**d**), respectively. In the images of (**a**–**d**), traces obtained for the last 12.6 s (6 frames × 2.09 s/frame) were superimposed with different colors for visualization purposes. Scale bar in (**a**) indicates 0.5 mm.

Figure 6 shows trace images obtained from the type B experiment for the top and bottom areas of the type B microchamber. The initial single cell in Figure 6 exhibited positive gravitaxis at the early stage of cell multiplication (Figure 6a) at day 0.04. After the first cell division, one of the two cells exhibited negative gravitaxis at day 0.78(Figure 6b), whereas another cell stayed at the bottom for cell division (not shown). This trend in gravitaxis change was the same as that observed in the type A microchamber for the cells with initially positive gravitaxis (Figure 5a). At a later stage of cell multiplication, at day 8.32, most of the cells exhibited negative gravitaxis (Figure 6d), whereas some showed positive gravitaxis at the bottom (Figure 6c). The band-like and circular traces evident in Figure 6c were due to the irregular movements of mitotic cells at the later stages of cell cleavage. As cell multiplication proceeded, the number of cells at the top area of the type B microchamber increased (Figure 6f), whereas an accumulation of immobile cells was observed at the bottom (Figure 6e). The immobile cells could not be detected by swimming traces (Figure 3i) but gradually accumulated and formed a stagnant layer at the bottom of the microchambers.

**Figure 7:**
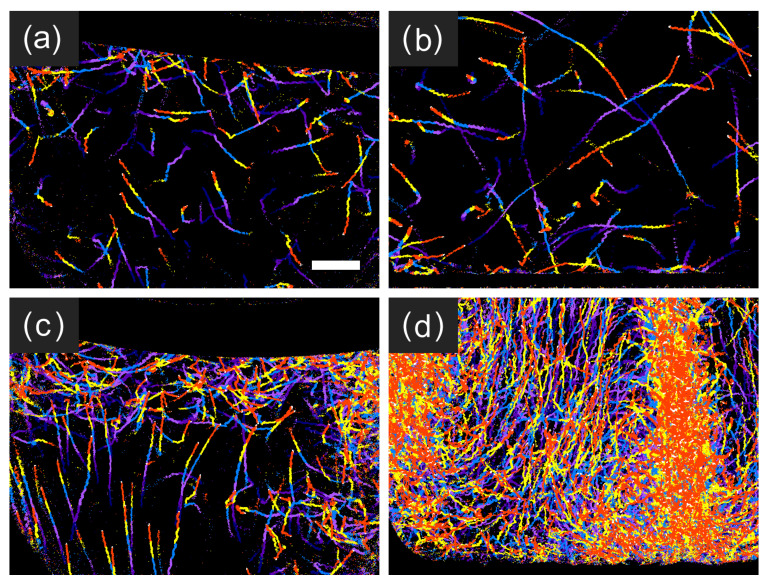
Trace image observed for type C experiment: (**a**) top area at day 1.00, (**b**) bottom area at day 0.93, (**c**) top area at day 4.11, and (**d**) bottom area at day 3.78. Black top area in (**a**,**c**) was air at the surface, black bottom area in (**b**,**d**) was the bottom of the type C chamber, and left bottom black corner in each image was due to the microscope’s observation edge. Traces for 12.4 s (6 frames × 2.06 s/frame) were superimposed with different colors for visualization purposes. Scale bar in (**a**) indicates 0.5 mm.

When the water column became larger and thicker in the type C chamber, a convectional flow was detected in the swimming traces. At the early stage of cell multiplication in the type C chamber (Figure 7a,b), observed swimming traces were randomly oriented, as is usual for *E. gracilis* in still water in a microchamber. However, the collective movements of the cells were observed after a couple of days as traces with the same direction at the surface (Figure 7c) and at the bottom (Figure 7d). Real-time cell movements are presented as Supplementary Movie S3 and S4 for the surface and bottom, respectively. The collective movements were due to convectional flow in which the cells were captured and flowed. The convection was not observed when the cell density was small, indicating that the convection was not thermal but was due to bioconvection [25,26,27]. Large numbers of cells gathering at the surface by negative gravitaxis produce a dense region. Because *E. gracilis* cells are denser than the CM medium [18], gyrotactic instability occurs. The dense region then starts to sink downward (Figure 7c, right side), drawing more cells into the region. The downward flow of dense cells reaches the bottom and can be observed as a dense column of cells (Figure 7d, right side). A large number of cells were brought to the bottom by bioconvection, resulting in the reversal of the cell population at the surface and bottom; the cells exhibited negative gravitaxis, but the cell population was denser at the bottom than at the surface.

## 4. Discussion

The main findings of this study are as follows: (1) a specific trend of gravitaxis change in the initially positive gravitactic cells and (2) a high rate of cell multiplication of those cells. Finding (1) revealed that cells transferred to fresh culture medium change their gravitaxis from initially positive to negative, then subsequently to moderate, and finally to negative in synchrony with cell multiplication. Whether those cells originally exhibited positive gravitaxis before transferring to fresh culture medium is not certain; however, it is probable that they exhibited negative gravitaxis before transferring, given that the majority of swimming cells after 4 days of culture showed negative gravitaxis (Figure 6d compared with Figure 6c). Therefore, some cells (ca. 30% in our experiments) switched from negative to positive gravitaxis upon transfer to fresh culture medium and started the gravitactic transition mentioned above as cell multiplication proceeded.

Finding (2) indicated that transfer to fresh culture medium induced a higher rate of cell multiplication only for those cells that switched their gravitaxis from negative to positive at transfer. This observation implies that cell multiplication is suppressed in cells after prolonged culture and that suppression is released by transfer to fresh culture medium. From findings (1) and (2), we deduced that, when transferred to fresh culture medium, approximately 30% of *E. gracilis* cells switch their metabolic status which governs gravitaxis and cell multiplication to initially positive gravitaxis with a high rate of cell multiplication.

Finding (1) also strongly suggests that the gravitaxis of *E. gracilis* is actively controlled by a previously unidentified flagellar motion and not passively by buoyancy, as imagined from the circular swimming traces [5,18], and some cells were observed to change their gravitaxis upon transfer to fresh culture medium. It appears more likely that the cells changed their gravitaxis by the active control of flagellar motion due to sensing a sudden environmental change rather than by changing body weight from stern-heavy to anterior-heavy. In addition, circular swimming traces were found in a few cases of the phototaxis of *E. gracilis* SM-ZK strain (Supplementary Figure S5). *E. gracilis* WT cells usually change their swimming direction in an on-site rotation manner by phototaxis, as we previously reported [28,29,30]. The majority of SM-ZK cells also show on-site rotation in phototaxis, but some cells exhibit circular swimming to turn toward a darker area. Despite rare cases in phototaxis, the observations suggest that *E. gracilis* possesses three modes of flagellar motion: straightforward swimming, on-site rotation, and circular swimming. The preferred mode of change to swimming direction for phototaxis is on-site rotation and, for gravitaxis, circular swimming. Compared with the photosensor in *E. gracilis* [31,32,33], the gravity sensor is believed to produce only occasional and weak signals [34,35], which may not be sufficient to induce on-site rotation but enough to induce only circular swimming.

The ecological significance of the change in gravitaxis over the time course of cell multiplication after transferring to fresh culture medium is unclear at present. It may simply be a side effect of switching metabolic status to higher cell multiplication, or it may contribute to survival by increasing diversity. Cell metabolism after transfer to fresh culture medium markedly differs among cells, and the initial gravitaxis appears be a good index to identify cells with a higher rate of multiplication.

## 5. Conclusions

The present study revealed temporal changes in the gravitaxis of *E. gracilis* cells after transfer to fresh culture medium. Approximately 30% of the transferred cells showed positive gravitaxis, which subsequently became negative, then moderate, and finally settled as negative. The trend of gravitaxis change was common among cells with initially positive gravitaxis, and such cells exhibited a higher rate of multiplication than those with initially negative gravitaxis. These findings suggest that, following transfer to fresh culture medium, approximately 30% of *E. gracilis* cells switch their metabolic status, which governs gravitaxis and cell multiplication, to initially positive gravitaxis with a higher rate of multiplication. The circular swimming observed for gravitaxis can be attributed to the active control of the flagella rather than to a passive buoyancy effect. The occurrence of bioconvection was also visualized as a vertical motion of cell mass. Our experimental method using a microchamber with single-cell confinement is an effective means of quantitatively investigating and analyzing gravitaxis and cell multiplication. Moreover, the method enables the visual determination of swimming motion and bioconvection.

## Supplementary Materials

The following are available online at https://www.mdpi.com/article/10.3390/plants10071411/s1, Figure S1: Initial stage of temporal change of the center of cell distribution (Xc, Yc), swimming activity evaluated as a TM value, and cell number estimated by our analyzing algorithm (initial part of Figure 2). Figure S2: Temporal changes in the center of cell distribution Yc and swimming activity evaluated as a TM value for cases of initially moderate gravitaxis. 14 cases are divided into (a) and (b). Movie S3: Real-time movie of 20 s at the top surface area in type C experiment at 3.78 day. A large number of cells are sinking down as a bioconvection column at the right side. The scene corresponds to Figure 7c (separate experiment). Movie S4: Real-time movie of 20 s at the bottom area in type C experiment at 3.78 day. A downward bioconvection column can be observed at the right side. The scene corresponds to Figure 7d (same experiment). Figure S5: Phototactic motions observed for SM-ZK cells. The microchamber was placed horizontally, and blue light (465–475 nm) of an intensity of 9.1 mW/cm2 was illuminated from the left side. Traces for 7.7 s (6 frames × 1.29 s/frame) were superimposed with different colors for visualization purpose. Scale bar in (a) indicates 0.5 mm.
