# Supplementary material for: Temporal Evolution of the Gravitaxis of Euglena gracilis from a Single Cell
Source: Plants (Basel). 2021 Jul 9;10(7):1411. doi: 10.3390/plants10071411 (PMC8309284; doi:10.3390/plants10071411)
Supplement: Supplementary file 1 [file plants-10-01411-s001.zip › paper_67_Supplementary.pdf]

Supplementary Figures with captions

Supplementary Fig. S1

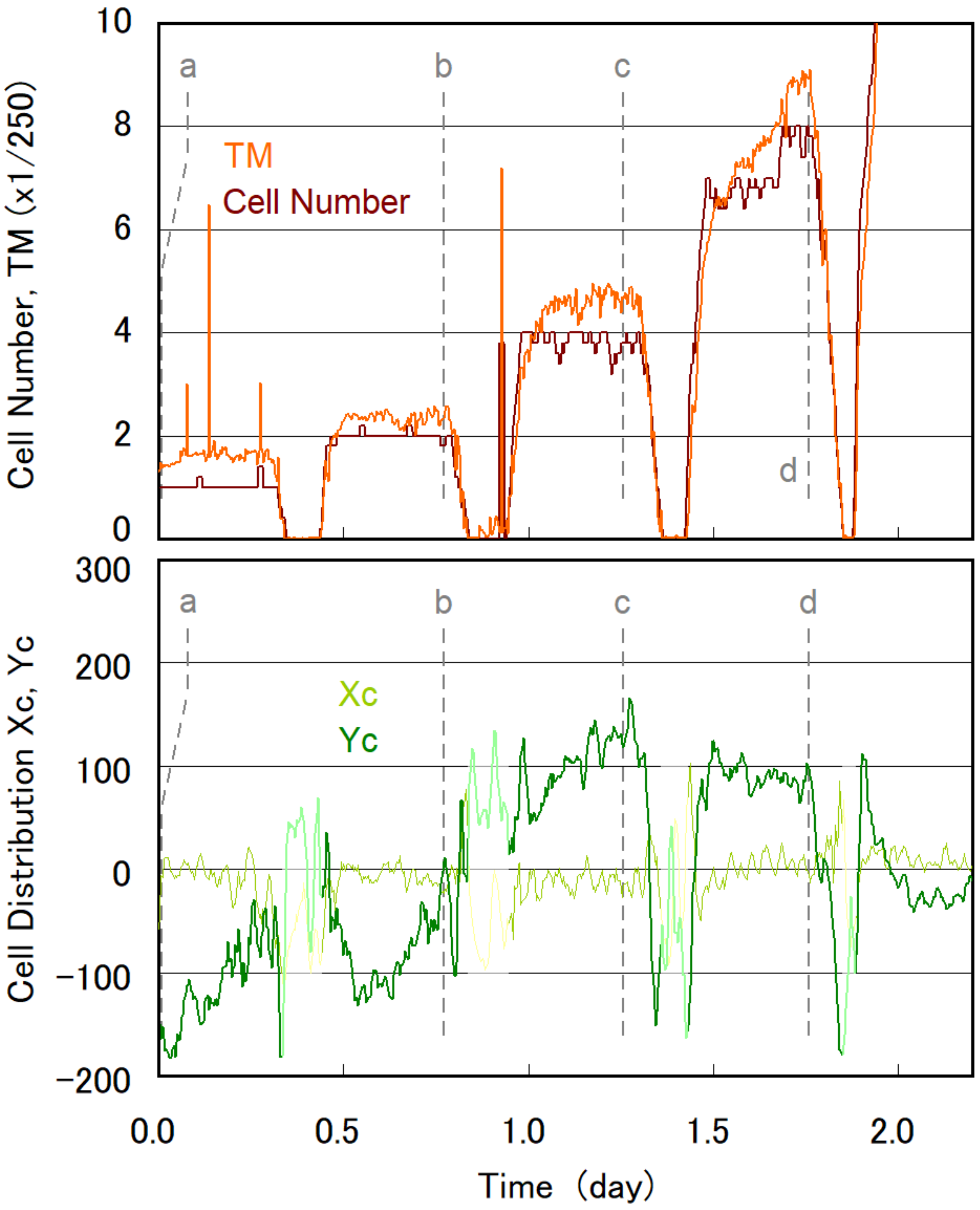

Initial stage of temporal change of the center of cell distribution (Xc, Yc), swimming activity evaluated as a TM value, and cell number estimated by our analyzing algorithm (initial part of Figure 2). Cell distribution Xc, Yc was not valid for the periods of no TM value, and faded in the figure. Irregular notches were due to some artifacts, such as noise and unintentional microchamber movements.

Fig. S2

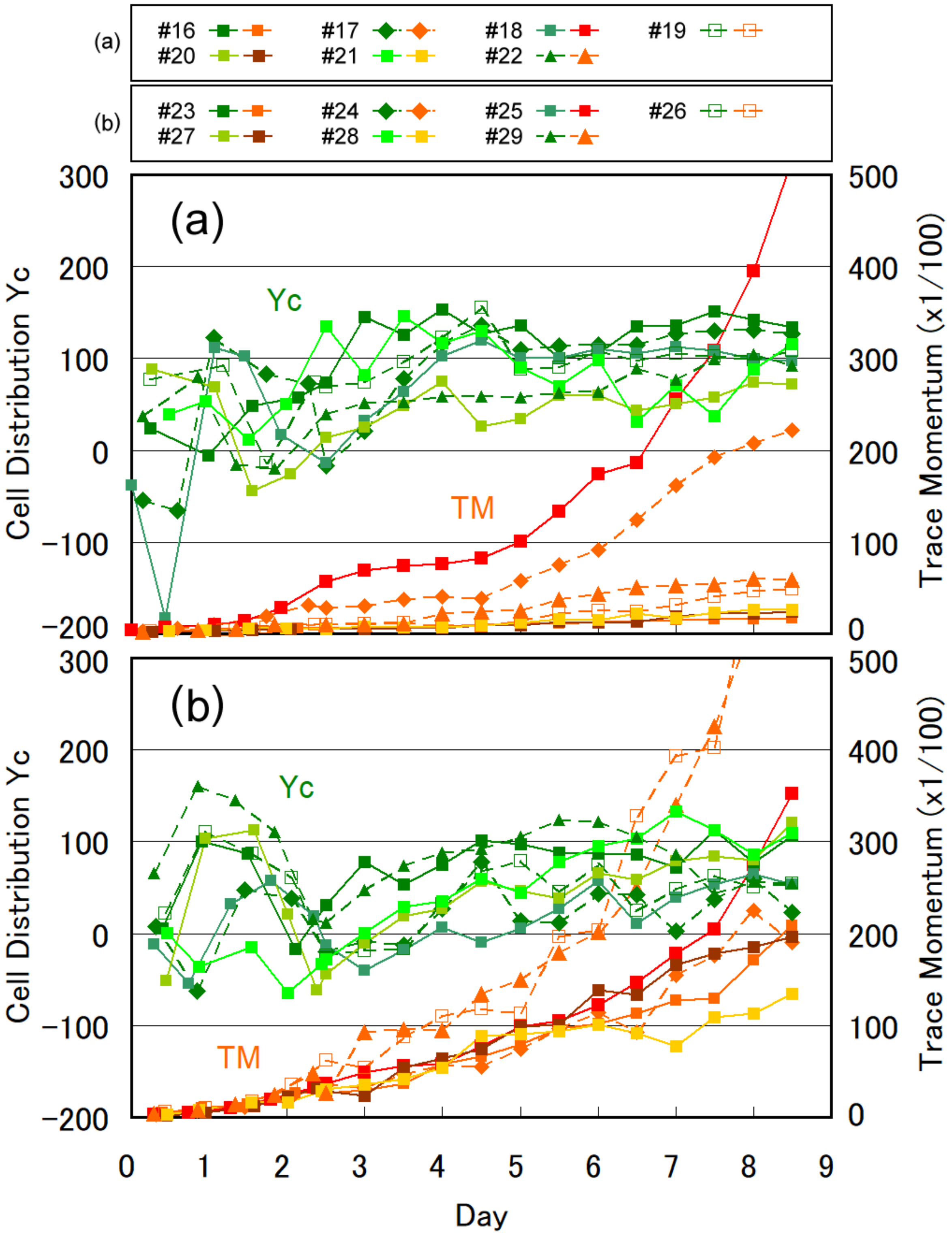

Temporal changes in the center of cell distribution Yc and swimming activity evaluated as a TM value for cases of initially moderate gravitaxis. 14 cases are divided into (a) and (b).

Movie S3, supplied by separate file Movie_67_S3.wmv

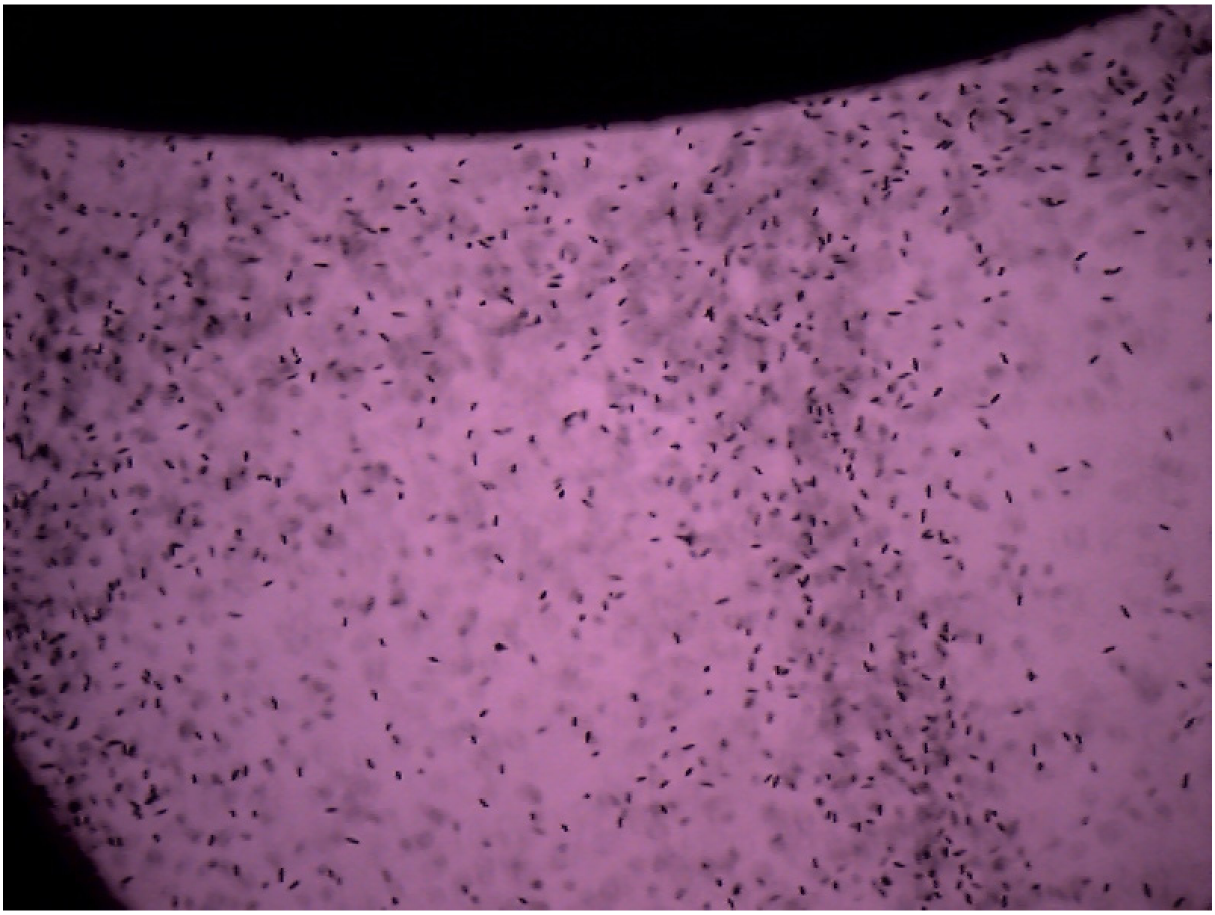

Real-time movie of 20 s at the top surface area in type C experiment at day 3.78. A large number of cells are sinking down as a bioconvection column at the right side. The scene corresponds to Figure 7c (separate experiment).

Movie S4, supplied by separate file Movie_67_S4.wmv

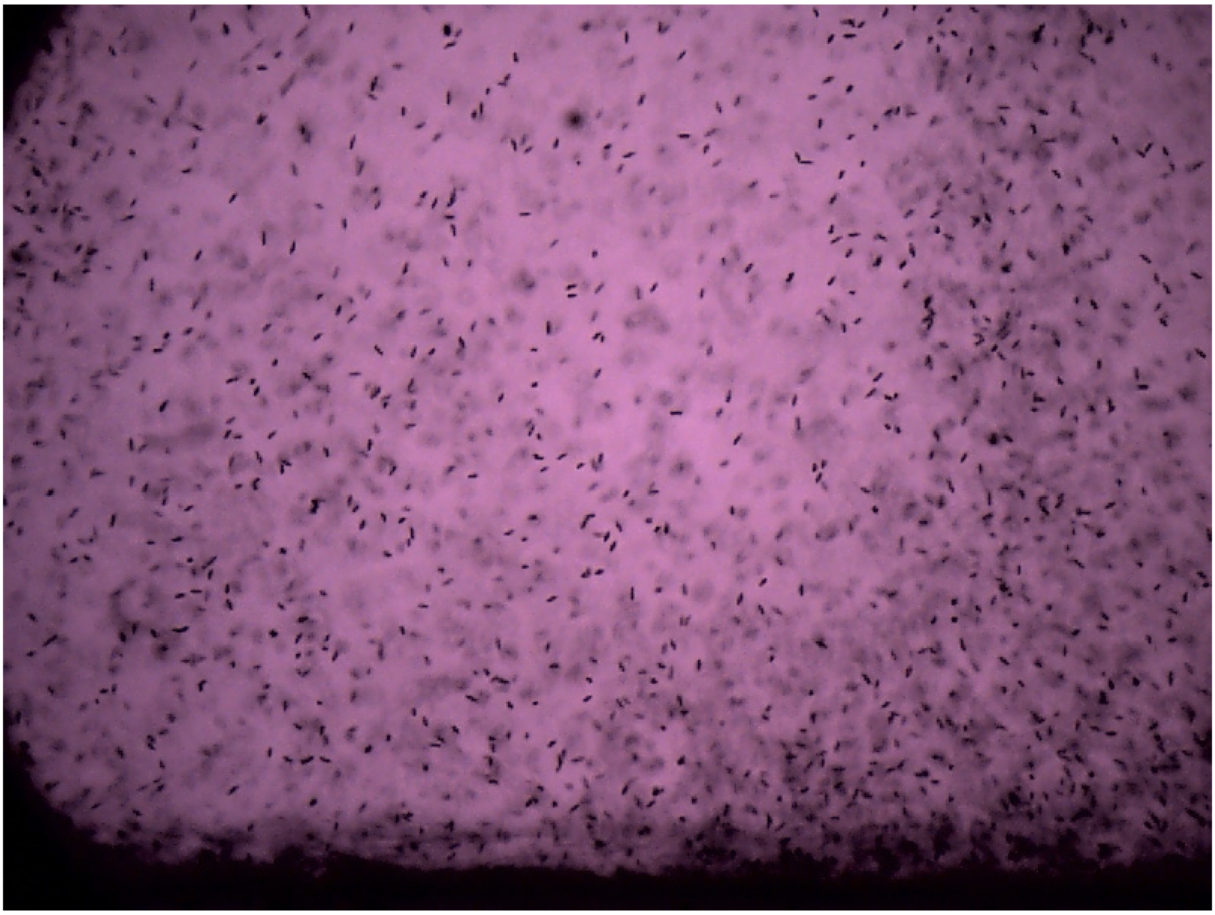

Real-time movie of 20 s at the bottom area in type C experiment at day 3.78. A downward bioconvection column can be observed at the right side. The scene corresponds to Figure 7d (same experiment).

Fig. S5

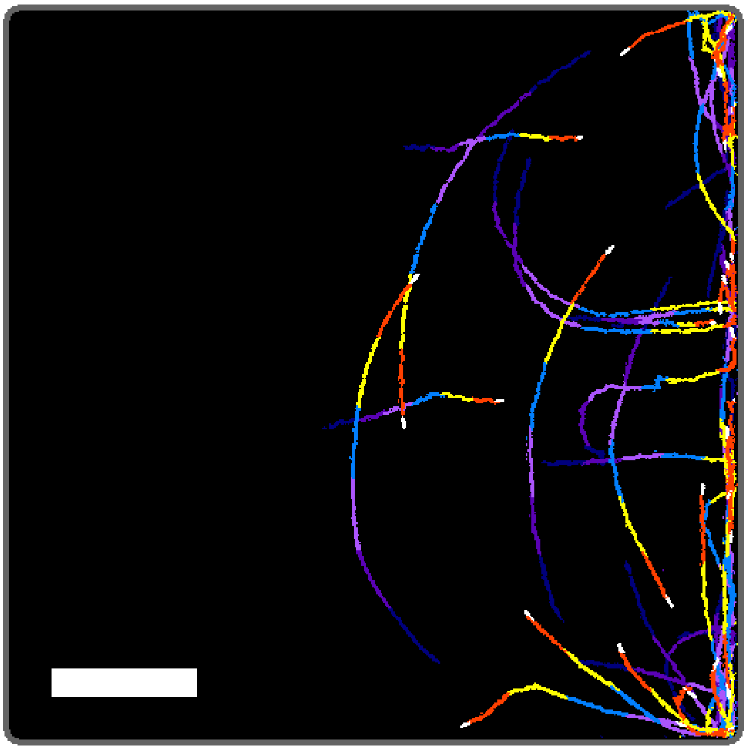

Phototactic motions observed for SM-ZK cells. The microchamber was placed horizontally, and blue light (465–475 nm) of an intensity of 9.1 mW/cm^2 was illuminated from the left side. Traces for 7.7 s (6 frames × 1.29 s/frame) were superimposed with different colors for visualization purpose. Scale bar indicates 0.5 mm.
